# Recovery of NanoLuc Luciferase-Tagged Canine Distemper Virus for Facilitating Rapid Screening of Antivirals *in vitro*

**DOI:** 10.3389/fvets.2020.600796

**Published:** 2020-12-10

**Authors:** Fuxiao Liu, Qianqian Wang, Yilan Huang, Ning Wang, Youming Zhang, Hu Shan

**Affiliations:** ^1^College of Veterinary Medicine, Qingdao Agricultural University, Qingdao, China; ^2^State Key Laboratory of Microbial Technology, Shandong University, Qingdao, China

**Keywords:** reverse genetics, rCDV-NLuc, luminescence, anti-CDV, high-throughput screening

## Abstract

Canine distemper virus (CDV), belonging to the genus *Morbillivirus* in the family *Paramyxoviridae*, is a highly contagious pathogen, affecting various domestic, and wild carnivores. Conventional methods are too cumbersome to be used for high-throughput screening of anti-CDV drugs. In this study, a recombinant CDV was rescued using reverse genetics for facilitating screening of anti-CDV drug *in vitro*. The recombinant CDV could stably express the NanoLuc® luciferase (NLuc), a novel enzyme that was smaller and “brighter” than others. The intensity of NLuc-catalyzed luminescence reaction indirectly reflected the anti-CDV effect of a certain drug, due to a positive correlation between NLuc expression and virus propagation *in vitro*. Based on such a characteristic feature, the recombinant CDV was used for anti-CDV assays on four drugs (ribavirin, moroxydine hydrochloride, 1-adamantylamine hydrochloride, and tea polyphenol) *via* analysis of luciferase activity, instead of *via* conventional methods. The result showed that out of these four drugs, only the ribavirin exhibited a detectable anti-CDV effect. The NLuc-tagged CDV would be a rapid tool for high-throughput screening of anti-CDV drugs.

## Introduction

Canine distemper (CD) is a highly contagious disease, affecting a wide variety of domestic and wild carnivores (1). Its typical signs include vomiting, diarrhea, dehydration, excessive salivation, coughing and/or labored breathing, loss of appetite, and weight loss. The respiratory, gastrointestinal, integumentary, and central nervous systems are most commonly affected in all species (2). The etiological agent of CD is canine distemper virus (CDV), which has been renamed canine morbillivirus, according to the latest virus taxonomy of the International Committee on Taxonomy of Viruses. The CDV is classified into the genus *Morbillivirus* in the family *Paramyxoviridae*, and its genome is a single strand of RNA with negative polarity, which encodes six structural (N, P, M, F, H, and L) and two nonstructural (V and C) proteins.

Commercially available CDV vaccines can confer protective immunity *in vivo*, whereas outbreaks of CD are still frequently reported in domestic and wild animals (3). Both practitioners and dog owners have a strong demand for effective anti-CDV drugs, nevertheless still unavailable to date. Dogs with early-stage CDV infection can be passively immunized with hyperimmune sera, which however only confer limited therapeutic effects on them. The purine nucleoside analog ribavirin (RIB) has a broad-spectrum activity against viruses (4), including CDV (5–7). Other drugs, like boceprevir (5), caffeic acid (8), and EICAR (9), have also been proven to possess anti-CDV characteristics. However, all of these drugs have not been approved for clinical therapy of CD.

There is a time- and labor-consuming process for the conventional screening of anti-CDV drugs *in vitro*. Taking the RIB as an example, the MTT assay would be firstly used to measure its 50% cytotoxic concentration (CC_50_), namely its concentration that reduces the viability of cells to 50% of the control. Subsequently, its 50% effective concentration (EC_50_) against CDV is measured by the 50% tissue culture infective dose (TCID_50_) assay alone or combined with other quantitative methods, e.g., qRT-PCR. Its anti-CDV Effectiveness can be finally described in terms of the selectivity index (SI, equal to CC_50_/EC_50_). The complete process is too cumbersome for the conventional method to be used for rapid screening of anti-CDV drugs.

Reverse genetics technique is widely used for the construction of recombinant CDVs to express foreign proteins, including the luciferase (10), an ideal reporter whose expression level generally implies the kinetics of viral replication. NanoLuc® luciferase (NLuc) is a novel small enzyme (19.1 kDa) engineered for optimal performance as a luminescent reporter. The luminescence catalyzed by the NLuc is about 150-fold brighter than that catalyzed either by firefly luciferase or by *Renilla reniformis* luciferase. The NLuc has been used as a better reporter to construct recombinant viruses for imaging and antiviral assays (11–14). In this study, to facilitate drug screening *in vitro*, an NLuc-tagged CDV was rescued, identified, characterized, and finally used for anti-CDV assays on four drugs, namely RIB, moroxydine hydrochloride (MOR), 1-adamantylamine hydrochloride (AMA) and tea polyphenol (TP).

## Materials and Methods

### Cells and Virus

The BSR-T7/5 and Vero-Dog-SLAM (VDS) cells, kindly provided by the China Animal Health and Epidemiology Center, were cultured at 37°C with 5% CO_2_ in Dulbecco's modified Eagle's medium (DMEM) supplemented with 10% fetal bovine serum (FBS), and containing penicillin (100 U/mL), streptomycin (100 μg/mL), amphotericin B (0.25 μg/mL), and G418 (500 μg/mL). A wild-type CDV (wt-CDV), QN (Qing Nong) strain, was propagated in VDS cells.

### Construction of Plasmids

**Figure 1:**
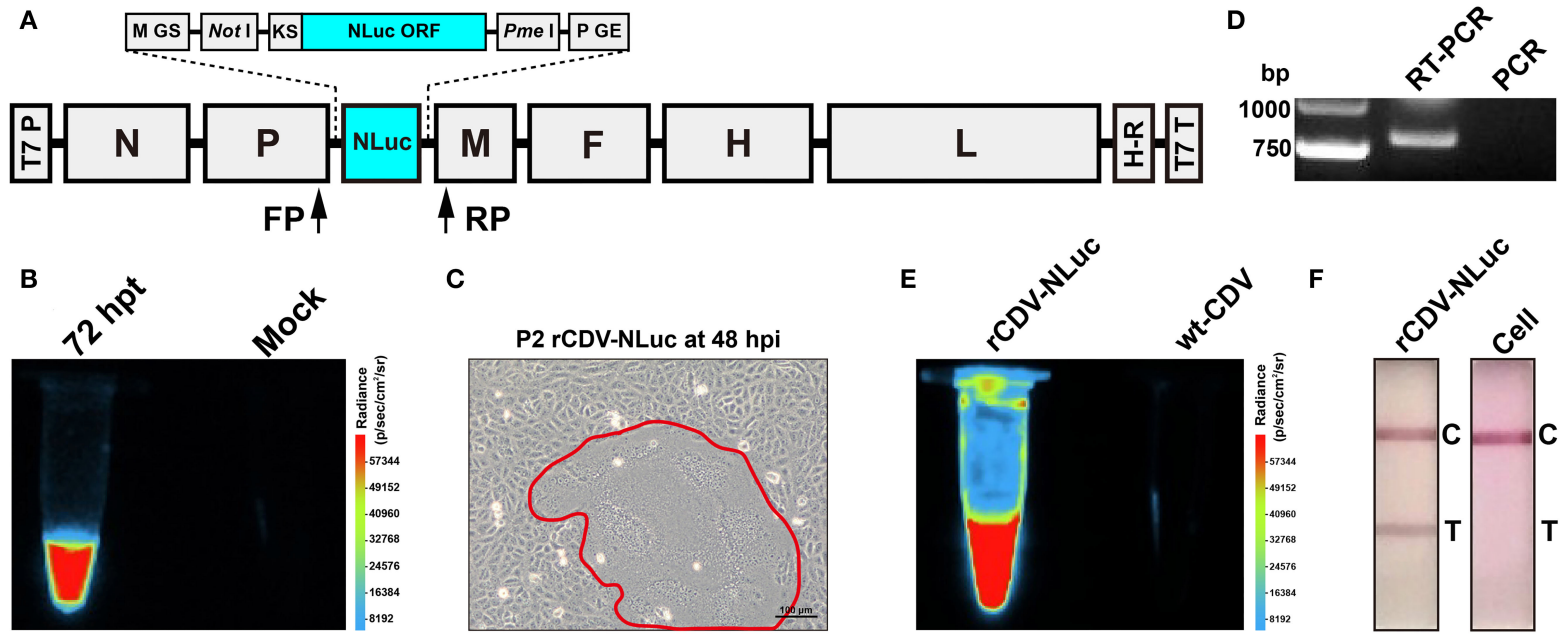
Construction and identification of the rCDV-NLuc. Schematic representation of rCDV-NLuc cDNA clone **(A)**. GS: gene start; GE: gene end; KS: Kozak sequence; NLuc ORF: NanoLuc® luciferase open reading frame; T7 P: T7 promoter; H-R: hepatitis delta virus ribozyme; T7 T: T7 terminator; FP: forward primer; RP: reverse primer. Luminescence assay of BSR-T7/5 cells co-transfected with four plasmids **(B)**. Syncytium formation (encircled by closed line) on a VDS cell monolayer infected with the P2 rCDV-NLuc at 48 hpi **(C)**. RT-PCR and PCR analyses of the P5 rCDV-NLuc using the FP/RP primers **(D)**. Luminescence assays of the P6 rCDV-NLuc and the wt-CDV **(E)**. Detection of rCDV-NLuc- and non-infected cells by test strips **(F)**. T, test; C, control.

The genome sequence of CDV 5804P strain (Genbank access No.: AY386316) was used to design a cDNA clone (Figure 1A) for rescuing the NLuc-tagged recombinant CDV (rCDV-NLuc). This cDNA clone was regulated by the T7 promoter at its 5′ end; at its 3′ end, a hepatitis delta virus ribozyme sequence was introduced between it and a T7 terminator sequence. The NLuc open reading frame (ORF) (Genbank access No.: JQ513379) was flanked by the Kozak sequence (15) at its 5′ end to improve NLuc expression. The Kozak sequence-NLuc ORF was flanked by *Not* I and *Pme* I recognition sites at its 5′ and 3′ ends, respectively. The modified NLuc ORF was regulated by M gene start (GS) and P gene end (GE) sequences at its 5′ and 3′ ends, respectively. The rCDV-NLuc cDNA clone was chemically synthesized and subcloned into the pBR322 plasmid. Three ORFs coding for N, P and L proteins were separately subcloned into an expression vector, pCAGGS. Three recombinant plasmids, pCAGGS-N, pCAGGS-P, and pCAGGS-L, served as helper plasmids for rescuing the rCDV-NLuc.

### Rescue of rCDV-NLuc

To rescue the rCDV-NLuc, BSR-T7/5 cells were co-transfected with the rCDV-NLuc cDNA clone and three helper plasmids, as described previously (16). Either of co-transfected cell monolayers was subjected to one freeze-and-thaw cycle at 72 h post transfection (hpt) for luminescence assay using the Nano-Glo® Luciferase Assay System (Promega, Madison, WI). The Fusion FX image analyzer (Vilber Lourmat, France) was used to observe luminescence of the co-transfected cell culture. The other co-transfected cell monolayer was digested with trypsin, and then co-cultivated with VDS cells in a T75 flask. The rCDV-NLuc would be recovered and released from the BSR-T7/5 cells, consequently infecting the VDS cells. The rCDV-NLuc was subjected to serial blind passages in VDS cells.

### RT-PCR Analysis of rCDV-NLuc

The culture supernatant of rCDV-NLuc at passage-5 (P5) was harvested for extraction of viral RNA, which was used as template for RT-PCR analysis using the PrimeScript™ One Step RT-PCR Kit (Takara, Dalian, China). The forward primer (FP: 5′-gatcaaaagtatcacacatgcttaa-3′) targeted the downstream region of P ORF, and the reverse primer (RP: 5′-gatcgaagtcgtacacctcagtcat-3′) targeted the upstream region of M ORF (Figure 1A). The RT-PCR reaction underwent 50°C for 30 min, 94°C for 2 min and then 30 cycles at 94°C (30 s), 55°C (30 s), and 72°C (50 s) in a thermocycler. The extracted RNA was simultaneously subjected to PCR analysis as a control using the same primers. The PCR reaction contained 2 × PrimeSTAR Max Premix (Takara, Dalian, China) and underwent 35 cycles at 98°C (10 s), 55°C (10 s), and 72°C (10 s). RT-PCR and PCR products were detected by agarose gel electrophoresis, followed by Sanger sequencing for analyzing the RT-PCR product.

### Luminescence Assay of rCDV-NLuc

Culture supernatant of the P6 rCDV-NLuc was subjected to luminescence assay as described in Subheading “Rescue of rCDV-NLuc,” and that of the wt-CDV was simultaneously analyzed as a control.

### Test Strip Detection of rCDV-NLuc

Culture supernatant of the P6 rCDV-NLuc was subjected to detection by a test strip (BIOKON^TM^), according to the manufacturer's instruction, and that of non-infected cells was simultaneously analyzed as a control.

### Growth Curve of rCDV-NLuc

Growth kinetics of the P7 rCDV-NLuc was compared with that of the wt-CDV *in vitro*. Briefly, VDS cells were plated into five 12-well plates (1.5 × 10^6^ cells/well, and 6 wells/plate) for incubation at 37°C for 2 h. The rCDV-NLuc and wt-CDV was separately inoculated (MOI = 0.001) into all plates (3 wells/sample) for incubation at 37°C for 2 h, and then supernatants were replaced with DMEM for further incubation at 37°C. At 0, 24, 48, 72, and 96 h post infection (hpi), any of plates was randomly removed from the incubator, and subjected to one freeze-and-thaw cycle to collect supernatant for viral titration by TCID_50_ assay. The viral titer for each sample was calculated by the Spearman-Kärber equation (17).

### Kinetics of NLuc Expression

VDS cells were plated into five 12-well plates (7 × 10^5^ cells/well, and 3 wells/plate) for incubation at 37°C for 2 h. The rCDV-NLuc was inoculated into all plates (MOI = 0.001) for incubation at 37°C for 4 h, and then supernatants were removed. The wells were gently rinsed once with D-PBS, and supplemented with DMEM for further incubation at 37°C. At 0, 24, 48, 72, and 96 hpi, any of plates was randomly removed from the incubator and subjected to two freeze-and-thaw cycles to collect supernatant, subsequently diluted 100-fold with PBS for luminescence assay in a black 96-well plate using the Tecan microplate reader.

### Genetic Stability of NLuc

The rCDV-NLuc was serially passaged in VDS cells for fifteen generations. Luminescence corresponding to its progenies (P5 to P15) was observed using the Fusion FX image analyzer. The culture supernatants at P7, P9, P11, P13, and P15 were harvested to extract viral RNAs for RT-PCR analysis using the FP/RP primers, as described in Subheading “RT-PCR Analysis of rCDV-NLuc.” Five RT-PCR products were detected by agarose gel electrophoresis, and subsequently subjected to Sanger sequencing.

### rCDV-NLuc-Based Antiviral Assays

Four drugs, RIB (Solarbio, Beijing, China), MOR (Sangon, Shanghai, China), AMA (Sangon, Shanghai, China), and TP (MACKLIN, Shanghai, China), were separately measured for determining their anti-rCDV-NLuc effects *in vitro*. The MTT assay was used to determine VDS cell viability as described previously (18). Briefly, stock solution of a given drug was serially diluted 2-fold with DMEM (Table 1). VDS cells were seeded in a 96-well plate at a density of 2 × 10^4^ cells/well, and incubated at 37°C for 2 h. Supernatant was replaced with the DMEM that contained the serially diluted drug solution, five replicate wells per dilution. The drug-treated cells were incubated at 37°C for 48 h. Subsequently, MTT solution was added to each well in a final concentration of 0.5 mg/mL, and then the 96-well plate was incubated at 37°C. After 4 h incubation, supernatants were replaced with dimethyl sulfoxide (DMSO, 100 μL/well) to dissolve violet formazan crystals. The plate was read using a microplate reader at 570 nm wavelength. The CC_50_ value for a given drug was calculated by nonlinear regression fitting using the GraphPad Prism software (Version 7.0).

**Table 1:** Drugs subjected to serial 2-fold dilutions for measuring their CC_50_, EC_50_, and SI values.

**Drugs**	**Dilutions for measuring CC_**50**_ value**	**CC_**50**_ value**	**Dilutions for measuring EC_**50**_ value**	**EC_**50**_ value**	**SI value**
RIB	0, 128, 256, 512, 1024, 2048, 4096, 8192, and 16384 μM	6141 μM	0, 32, 64, 128, 256, 512, and 1024 μM	432 μM	14.2
MOR	0, 80, 160, 320, 640, 1280, 2560, 5120, and 10240 μM	3415 μM	0, 50, 100, 200, 400, 800, and 1600 μM	NA	NA
AMA	0, 40, 80, 160, 320, 640, and 1280 μM	585 μM	0, 4, 8, 16, 32, 64, 128, and 256 μM	NA	NA
TP	0, 0.25, 0.5, 1, 2, 4, 8, and 16 μg/mL	5.7 μg/mL	0, 0.125, 0.25, 0.5, 1, 2, and 4 μg/mL	NA	NA

*RIB, ribavirin; MOR, moroxydine hydrochloride; AMA, 1-adamantylamine hydrochloride; TP, tea polyphenol; NA, not available. CC_50_, 50% cytotoxic concentration; EC_50_, 50% effective concentration; SI, selectivity index (CC_50_/EC_50_)*.

Based on results of cytotoxicity assays on them, the four drugs were separately tested for their antiviral activities at different concentrations. Briefly, VDS cells were seeded in a 24-well plate at a density of 7 × 10^5^ cells/well, and incubated at 37°C for 2 h. Subsequently, the cells were inoculated with rCDV-NLuc (MOI = 0.001), and incubated at 37°C for 4 h. Supernatant was replaced with the DMEM that contained the serially diluted drug solution, three replicate wells per dilution. After incubation at 37°C for 48 h, the 24-well plate was subjected to two freeze-and-thaw cycles to collect supernatants for luminescence assay in a black 96-well plate using the Tecan microplate reader. Anti-rCDV-NLuc activity of a given drug was expressed as the EC_50_, calculated by nonlinear regression fitting using the GraphPad Prism software to determine the drug concentration required to achieve 50% of relative luminescence unit (RLU) reduction.

## Results

### Recovery of rCDV-NLuc

The co-transfected BSR-T7/5 cell monolayer was subjected to one freeze-and-thaw cycle at 72 hpt for collecting supernatant, which then was mixed with the furimazine substrate (Promega, Madison, WI). When analyzed using the Fusion FX image analyzer, the mixture emitted bright luminescence, but unobservable in that without co-transfection of helper plasmids (Figure 1B). The rescued rCDV-NLuc was serially passaged in VDS cells. A typical cytopathic effect (CPE), syncytium formation on a cell monolayer, was visible at 48 hpi during the second passaging (Figure 1C).

### Identification of rCDV-NLuc

Total RNA of the P5 rCDV-NLuc was analyzed by RT-PCR to confirm its identity. An expected band of amplicon size (799 bp) was observed only on the RT-PCR lane (Figure 1D). As a control, PCR analysis implied no cDNA clone residues affecting RT-PCR detection (Figure 1D). The Sanger sequencing showed that the P5-based RT-PCR product was identical to the 799-bp sequence. Culture supernatant of the P6 rCDV-NLuc, when mixed with the furimazine substrate, could still emit bright luminescence, nevertheless unobservable in that of the wt-CDV (Figure 1E). Meanwhile, rCDV-NLuc-infected and non-infected culture supernatants were independently detected by test strips, indicating a positive result only on the former (Figure 1F).

### Growth Curve of rCDV-NLuc

**Figure 2:**
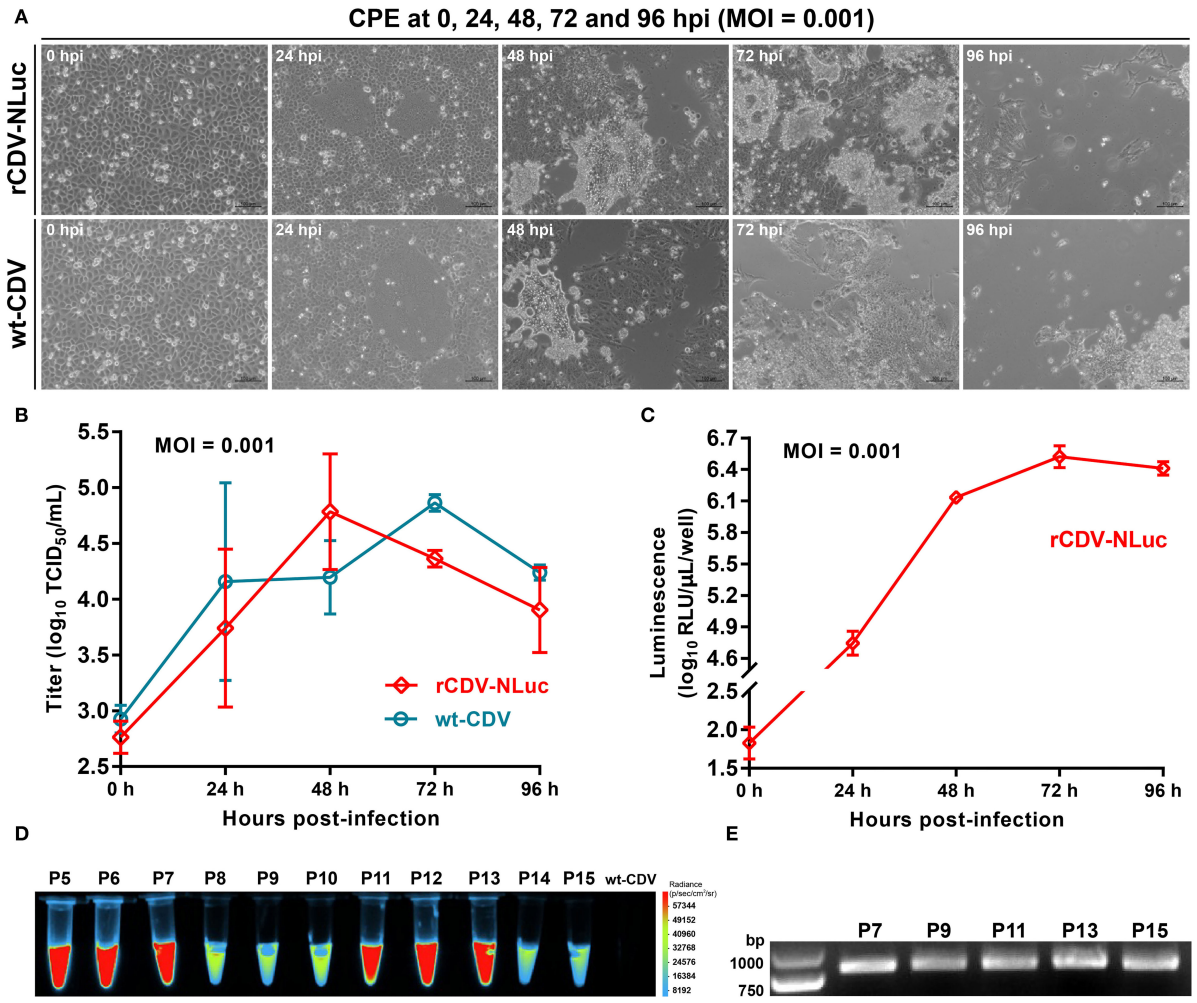
Characterization of the rCDV-NLuc. CPEs on VDS cell monolayers infected (MOI = 0.001) either with the P7 rCDV-NLuc or with the wt-CDV at 0, 24, 48, 72, and 96 hpi **(A)**. Multi-step growth curves of the P7 rCDV-NLuc and the wt-CDV in VDS cells during 96 hpi **(B)**. Kinetics of NLuc expression by the rCDV-NLuc in VDS cells during 96 hpi **(C)**. RLU: relative luminescence unit. Luminescence assay on genetic stability of the NLuc during serial virus passages in VDS cells **(D)**. RT-PCR analyses of the P7, P9, P11, P13, and P15 rCDV-NLucs using the FP/RP primers **(E)**.

Growth kinetics of the P7 rCDV-NLuc was compared with that of the wt-CDV during 96 hpi. Syncytia induced by both viruses were observable at 24 hpi, and exacerbated over time to form a phenotype of intercellular hyperfusogenicity at 48 hpi (Figure 2A). Both viruses exhibited different growth kinetics. The rCDV-NLuc replicated more slowly than the wt-CDV in VDS cells did except at 48 hpi (Figure 2B).

### Kinetics of NLuc Expression

To examine the kinetics of NLuc expression *in vitro*, VDS cells were infected with the rCDV-NLuc at MOI of 0.001, and luciferase activities were measured at each time point of infection. The kinetics curve showed that 0 to 48 hpi was an exponential phase of NLuc expression (Figure 2C), similar as that of the rCDV-NLuc growth curve within 48 hpi (Figure 2B). Despite higher rCDV-NLuc titer at 48 hpi than that at 72 hpi (Figure 2B), the NLuc expression reached its plateaus at 72 hpi (Figure 2C).

### Genetic Stability of NLuc

To test the stability of NLuc expression, the rCDV-NLuc was serially passaged in VDS cells for a total of fifteen passages. The NLuc expression was clearly detectable at each of passages (P5 to P15) through observation of luminescence (Figure 2D). The FP/RP-based RT-PCR showed that the 799-bp-specific product was amplified from individual RNAs corresponding to the P7, P9, P11, P13, and P15 viruses (Figure 2E). All RT-PCR products were subjected to Sanger sequencing, indicating that they were identical to the 799-bp sequence.

### rCDV-NLuc-Based Antiviral Assays

**Figure 3:**
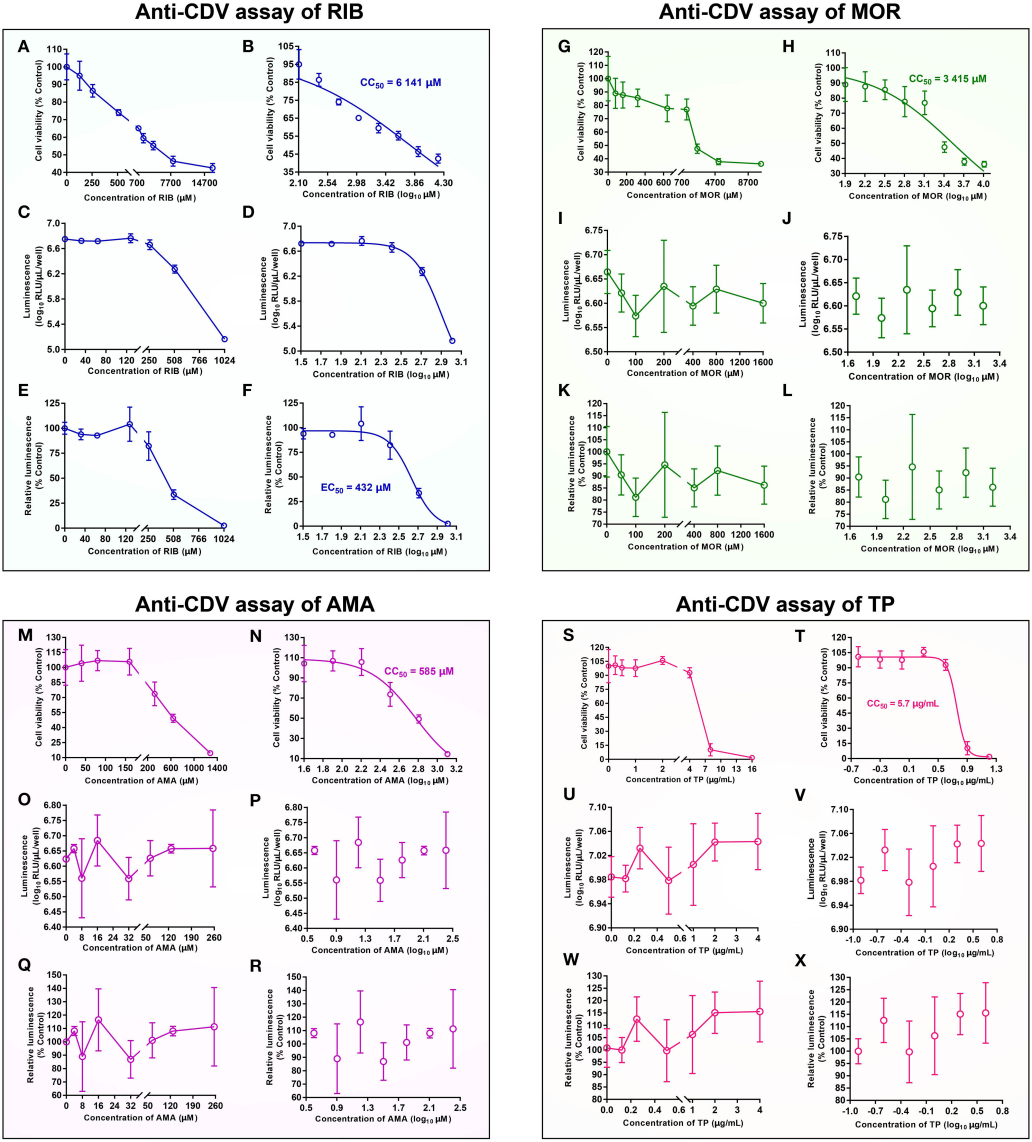
Anti-CDV analyses of ribavirin (RIB) **(A–F)**, moroxydine hydrochloride (MOR) **(G–L)**, 1-adamantylamine hydrochloride (AMA) **(M–R)** and tea polyphenol (TP) **(S–X)** *in vitro*. MTT assay on relative viability of VDS cells at 48 h after drug treatment at different concentrations **(A,G,M,S)**. The data represent the mean ± SD for five replicates per dilution. The CC_50_ value for a drug, calculated by nonlinear regression fitting using the GraphPad Prism software **(B,H,N,T)**. Variation of luminescence intensity in rCDV-NLuc-infected VDS cells at 48 h after drug treatment with different concentrations **(C,I,O,U)**. The data represent the mean ± SD for three independent experiments. The data in **(C,I,O,U)** are processed by nonlinear regression fitting using the GraphPad Prism software, and then shown in **(D,J,P,V)**. Variation of relative luminescence intensity in rCDV-NLuc-infected VDS cells at 48 h after drug treatment with different concentrations **(E,K,Q,W)**. The EC_50_ value for a drug against the rCDV-NLuc, calculated by nonlinear regression fitting using the GraphPad Prism software **(F,L,R,X)**.

The effects of four drugs on VDS cell viability were separately determined by the MTT assay (Figures 3A,G,M,S). Their CC_50_ values (Table 1) were calculated by nonlinear regression fitting using the GraphPad Prism software (Figures 3B,H,N,T). All drug-treated cell cultures were subjected to the luminescence assay at 48 hpi (Figures 3C,D,I,J,O,P,U,V). Their anti-rCDV-NLuc effects were quantified by determination of their EC_50_ values (Table 1) through nonlinear regression fitting using the GraphPad Prism software (Figures 3E,F,K,L,Q,R,W,X). The result showed that out of these four drugs, only the RIB exhibited a detectable anti-rCDV-NLuc effect in VDS cells.

When its concentration was <128 μM, the RIB exhibited no significant effect against the rCDV-NLuc, as evidenced by almost the same luminescence intensities with concentrations <128 μM. As the RIB concentration increased 2-fold, there were, however, gradual decreases in luminescence intensity, implying the NLuc expression that was gradually inhibited by the RIB (Figures 3C,D). The antiviral effect of RIB was quantitatively measured for determining its EC_50_ value, estimated at 432 μM (Figure 3F). The SI value (CC_50_/EC_50_) was 14.2 for the RIB against rCDV-NLuc in VDS cells.

A SI value ≥ 4 is generally considered suitable for an antiviral drug (19). In order to assess an effective EC_50_ value, the concentration range of a drug for antiviral screening should be limited to one quarter of its CC_50_ value. The luminescence intensity was not found decreasing gradually with increasing drug concentration in the MOR-, AMA- and TP-treated groups (Figures 3I,J,O,P,U,V). Their EC_50_ values could not be calculated by nonlinear regression fitting using the GraphPad Prism software (Figures 3K,L,Q,R,W,X).

## Discussion

The reverse genetics system for CDV had been established as early as 2000 (20). CDV is an ideal vector for expressing various foreign proteins, including green fluorescence protein (GFP) (21), red fluorescence protein (22), firefly luciferase (10), interleukin-7 (23), interleukin-18 (24), and rabies virus glycoprotein (25). The NLuc is a novel luciferase that has many physical properties, making it an excellent reporter protein (26). This prompted us to rescue the rCDV-NLuc for facilitating antiviral screening through analysis of NLuc expression.

In this study, a low-copy-number plasmid (pBR322) was used for constructing the rCDV-NLuc cDNA clone, into which the 5′- and 3′-end-modifying NLuc was subcloned. The rCDV-NLuc could be readily rescued from its cDNA clone, because of bright luminescence that was visible at 72 hpt or even earlier. The VDS cell line was susceptible to CDV infection, whereas the VDS cell-propagated rCDV-NLuc at 48 hpi only reached the average peak titer of 4.8 log_10_ TCID_50_/mL, approximately equal to that corresponding to the wt-CDV infection at 72 hpi. Another morbillivirus, peste des petits ruminants virus, when cultivated in the same cell line, can reach a peak titer of ~6.0 log_10_ TCID_50_/mL (16). The NLuc might not be prone to degradation in VDS cells, because the highest level of NLuc expression appeared at 72 hpi (Figure 2C) or even later, while the rCDV-NLuc titer had begun to decrease before 72 hpi (Figure 2B).

As to a certain recombinant virus that is able to express a heterologous protein, the genetic stability of foreign sequence is an easily overlooked but important aspect, which impacts on further application of the recombinant virus. Compared with some positive-sense and single-stranded RNA viruses (27, 28), from which non-self sequences can be easily deleted due to their genetic instabilities, paramyxoviruses are able to retain foreign sequences in their genomes with serial passaging in cells. We simultaneously constructed another recombinant CDV that contained a GFP marker, which could be stably expressed during serial passages even under the continuous selective pressure of mutagen. We also examined the genetic stability of NLuc during serial fifteen passages of the rCDV-NLuc by Sanger sequencing, showing neither point mutations nor fragment deletions in the NLuc sequence at the P7, P9, P11, P13, and P15.

As a proof-of-concept that the rCDV-NLuc was feasible for use as an antiviral screening tool, we assessed the effects of four common drugs. The RIB is a nucleoside analog of guanosine that may interfere with the synthesis of viral mRNA. It is primarily indicated for use in treating hepatitis C and viral hemorrhagic fevers (29, 30), and also has been proven to be an antiviral against CDV (5–7). We confirmed its anti-CDV effect *via* analysis of luciferase activity, instead of the conventional TCID_50_ assay. An antiviral effect is generally described in terms of the SI value, which ≥ 4 is generally considered suitable for an antiviral drug (19). It was 14.2 for RIB against CDV in this study, confirming that the RIB was an ideal anti-CDV drug with a relatively safe profile. Besides the RIB, we identified other two drugs with anti-CDV activity using this novel method and the conventional TCID_50_ assay (data not shown). The MOR is a synthetic antiviral compound that chemically belongs to the series of the heterocyclic biguanidines, and was originally developed in the 1950s as an influenza treatment (31). AMA and TP are also effective drugs against the influenza virus (32, 33). However, these three drugs were unable to inhibit the rCDV-NLuc replication in this study, as evidenced by their individual luminescence assays (Figure 3).

The TCID_50_ assay, albeit commonly used for screening of anti-CDV drugs, is a time- and labor-consuming method. On the one hand, it generally lasts for 5 to 7 days or longer to observe CPE on cell monolayers for the calculation of CDV titer. On the other hand, use of TCID_50_ assay would be very cumbersome to measure an EC_50_ value of drug. Any of CDV-infected cultures would be titrated independently for three replicates using three 96-well plates. If the TCID_50_ assay had been used in this study, we would have used twenty-one (7 drug concentrations × 3 replicates) 96-well plates, nonetheless only enough to measure one EC_50_ value for a given drug. Therefore, the TCID_50_ assay is not a rapid tool for high-throughput drug screening. Other common methods for quantitative assays include qRT-PCR and Western blot (34), both of which can be completed within 1 to 2 days, whereas also labor-consuming due to intra-references required for quantitative analysis.

In order to establish a novel method, we constructed the rCDV-NLuc, which greatly shortened a period of anti-CDV assay. The luminescence-based EC_50_ assay on one drug can usually be completed within only one hour. There was a positive correlation between the NLuc expression and the rCDV-NLuc propagation *in vitro* within 48 hpi (Figures 2B,C). Thus, the luminescence intensity can indirectly reflect the anti-CDV effect of a certain drug within 48 hpi. This is the reason why the rCDV-NLuc-based method has a high-throughput potential in drug screening. Moreover, intra-references are generally unnecessary for this method, further simplifying the procedure of drug screening. Additionally, the P7 rCDV-NLuc has been recently shown to be unable to induce typical signs in minks by a preliminary experiment (data not shown), implying that this recombinant should be a virulence-attenuated strain, which would lower a potential risk of bio-safety, when used for screening of antivirals. Despite many advantages over the conventional methods, a major limitation of the novel tool is that drugs are unable to interfere with the NLuc expression and activity. Therefore, although this method is suitable to high-throughput screening (or rather, prescreening) of anti-CDV agents, the TCID_50_ assay still is the gold standard for quantifying anti-CDV effect of a given drug.

## Data Availability Statement

The original contributions presented in the study are included in the article/supplementary material, further inquiries can be directed to the corresponding author/s.

## Author Contributions

FL and QW: conceptualization and writing—original draft preparation. YH and NW: formal analysis. YZ and HS: project administration. All authors contributed to the article and approved the submitted version.

## Conflict of Interest

The authors declare that the research was conducted in the absence of any commercial or financial relationships that could be construed as a potential conflict of interest.
